# From internal echoes to sharing experiences: transformative learning with voice apps to enhance transgender care literacy in medical students

**DOI:** 10.3389/fpubh.2026.1767576

**Published:** 2026-03-09

**Authors:** Susanne Gahbauer, Shamim Sherafat, Michaela Wagner-Menghin

**Affiliations:** 1 Department of Social and Preventive Medicine, Center for Public Health, Medical University of Vienna, Vienna, Austria; 2 Department of Science and Technology Studies, University of Vienna, Vienna, Austria; 3 Vienna Institute of Demography, Austrian Academy of Sciences, Vienna, Austria; 4 Clinical Division for Social Psychiatry, Department of Psychiatry and Psychotherapy, Medical University of Vienna, Vienna, Austria; 5 Comprehensive Center for Clinical Neurosciences and Mental Health, Medical University of Vienna, Vienna, Austria

**Keywords:** medical education, narrative analysis, transformative learning, transgender care literacy, voice apps, voice care

## Abstract

**Introduction:**

Gendered voice perception is central to gender recognition and can be a major source of distress for transgender people, yet voice and voice therapy remain marginal in medical curricula. This study examined how a digital, experiential seminar using voice-feedback applications can foster medical students’ understanding of gendered voice and transgender health.

**Methods:**

We conducted a qualitative observational study within a 135-min online seminar, “Feminize Your Resonance!”, embedded in a fourth-year public health module at the Medical University of Vienna. One hundred medical students used commercially available voice apps to experiment with their own pitch and resonance and submitted app screenshots and written reflections via an online learning platform. We applied a narrative analysis, based on narrative episodes in students’ accounts, using a multi-stage coding process supported by qualitative data analysis software to map learning processes in several stages.

**Results:**

Analysis yielded three interlinked themes—implicit voice awareness, technology engagement, and sharing transgender experience—organized into a five-phase learning journey from pre-existing discomfort with one’s recorded voice to social awareness with voice challenges of transgender individuals. Students moved from internal, often negative, self-evaluation to critical appraisal of app algorithms, exploration of their own vocal plasticity, and perspective-taking on the challenges of gender-affirming voice transition.

**Conclusion:**

Technologically mediated voice exploration can catalyze transformative learning about the role of voice in gender incongruence and support the development of affective and clinical competencies in medical education. Potentially the training facilitates communicating with transgender individuals empathically, not only related to transgender-affirming care but in all healthcare settings.

## 1 Introduction

Pitch, volume, and resonance shape gendered voice recognition: high-pitched voices with brighter resonance and lower volume are typically perceived as feminine, whereas deeper pitch, darker resonance, and greater volume are associated with masculinity. Voice can therefore support one’s unique gender expression and enhance voice satisfaction (1). As Anne Carson (2) argues, voice serves as a central category for judging individuals in everyday interactions, and societies have long sought to classify voices within binary categories.

While pitch is a salient and often dominant cue in gender attribution, it does not operate in isolation. A substantial body of research demonstrates that gender perception in speech relies on a constellation of acoustic and prosodic features, including resonance, intonation, breathiness, articulation and speech dynamics, as well as linguistic and pragmatic cues. Importantly, voices within the overlapping pitch range typically described as gender-ambiguous (approximately 145–165 Hz) are not consistently perceived as such, indicating that pitch thresholds alone are insufficient to account for listener judgements (3, 4). Listener-based factors further complicate gender attribution, as individual experience, expectations, and sociocultural beliefs shape how vocal cues are interpreted (5). Taken together, these findings suggest that gendered voice perception emerges from the interaction of multiple, vocal parameters and listener perspectives rather than from pitch alone.

Gendered voice recognition can be particularly challenging for individuals living with “gender incongruence” (ICD-11). Since 2016, “gender incongruence” has been classified not as a mental health disorder but as a condition related to sexual health, defined as a “marked and persistent incongruence between an individual’s experienced gender and the assigned sex at birth” (ICD-11). In Austria, more than two-thirds of transgender individuals pursue gender-affirming hormone therapy, currently the most widely accessed form of gender-affirming care (6). Globally, the transgender population has increased to an estimated 0.3–0.5% of adults, with rising numbers of gender-affirming interventions (7).

As a recent study has empirically shown, voice is amongst the most important features contributing to gender incongruence, especially in social and professional interaction, where gender attribution is shaped by both vocal characteristics and listener interpretation (8). A consistent subjective perception of gender and voice go along with higher quality of life (9). General therapy options for voice alteration include behavioral, hormonal, and surgical voice interventions (10). Behavioral oriented voice therapy is non-invasive, bearing less risk compared to hormonal and surgical interventions, and is thus often favored as the first treatment for voice adjustment (11). As such, gender-affirming voice therapy is increasingly recommended and implemented for both transgender men and transgender women (12, 13).

Voice therapy nevertheless remains underrepresented in transgender healthcare. In an Austrian study, 82% of transgender individuals reported no access to voice therapy, frequently citing a lack of insurance coverage (6). The limited availability of in-person voice therapy has led to growing interest in digital applications designed to support voice-gender congruence (14). Apps such as Voice Analyst provide feedback on pitch, volume, resonance, and other vocal qualities, and are increasingly used by transgender individuals seeking to align their voice with their gender identity not only in Austria (15). This underscores the need to introduce medical and health professions (MHP) students to transgender-specific voice issues to support gender-affirming and technologically informed voice care.

Educating MHP students about transgender health issues, such as incongruence between visual and vocal appearance, is also important with respect to reducing discrimination in healthcare, as transgender individuals remain at increased risk of healthcare avoidance due to anticipated discrimination (16). To address knowledge, skills, and attitudes simultaneously, current teaching interventions for MHP students aiming at reducing discrimination in healthcare typically rely on intergroup contact through patient-educators (17, 18). In addition, simulation-based approaches aimed at fostering perspective taking and empathy have been shown to increase awareness of patients´ practical and emotional challenges (19).

As such, the present study posits that the exploration of one’s own voice, using voice-modulation technology, particularly technologically mediated feedback (TMF) enables learners, besides acquiring knowledge about voice therapy in transgender healthcare, to emotionally engage with their own vocal appearance. TMF, which can be provided immediately, can facilitate transformative learning (20). This study therefore seeks to describe how actively experimenting with voice apps to explore and reflect on one’s own gendered vocal expression as learning activity in a short seminar on voice and transgender health influence students’ empathizing with transgender individuals.

## 2 Methods

This qualitative study used an observational design and applied thematic analysis (21). This flexible narrative method allowed for a systematic examination of students´ reflections through their personal accounts (22) and is increasingly used in health services research to illuminate experiences that quantitative approaches cannot fully capture (23). We follow a pragmatist approach aimed at producing contextually relevant, literature-informed findings. To ensure methodological integrity, we adhered to the Standards for Reporting Qualitative Research (SRQR), a 21-item checklist addressing study context, methods, analysis, and reflexivity (24).

### 2.1 Educational setting

The online seminar *Feminize Your Resonance!* (135 min) engaged medical students with concepts related to gender and voice. Developed by the first author and refined after a 2024 pilot (n = 28), the seminar was offered as a mandatory elective (as one of nineteen comparable options), within the fourth-year public health module at the Medical University of Vienna. Currently the seminar is not offered in any other contexts.

Asynchronous mini-lectures (approx. 15 min each) introduced key content on how pitch, volume, and resonance shape gendered voice perception and how transgender individuals navigate gender-affirming voice transition. A final mini-lecture addressed voice assessment of voice characteristics and the potential role of apps in gender-affirming therapy. Materials included sound examples and a short film featuring a transgender woman discussing her therapeutic experience.

The seminar’s main component (90 min) comprised experiential learning framed as a “Gendered Voice Competition”. For this journey, students used a voice app (*Voice Analyst* or *Voice Tools*) to experiment with their voices and receive TMF. Both apps use algorithms based on pitch and volume and present data via percentages or colored visualizations indicating “female” and “male” portions of the voice sample. The app *Voice Analyst* further allows for non-binary voice ranges. It is widely used in clinical voice therapy and by transgender individuals but offered inconsistent free-trial access during the seminar period. *Voice Tools* was therefore offered as an alternative due to its consistent free-trial availability and the inclusion of English sentences and short texts for practice.

The instructor (first author) provided written guidance on app functions and examples of data outputs, supplemented by a synchronous Q&A. As the pilot study showed, offering a choice between synchronous and asynchronous participation was essential for participants to feel comfortable engaging in voice experimentation. Students then completed a self-directed *Gendered Voice Competition*, choosing from several practice suggestions designed-presented in Table 1 to encourage playful experimentation and disrupt habitual listening and speaking patterns.

**Table 1:** Practice suggestions in the *Gendered Voice Competition.*

	Practice suggestion
1	Experiment with the predefined English sentences and texts provided by Voice Tool, try how far you can modify the gendered voice region of your own voice.
2	Think of three of your favorite phrases your – presumably female-voiced - digital assistant, uses. Give your voice a male-sound while saying them.
3	Choose a (short) text. Give your voice as many feminine characteristics as possible while reading the text.
4	Try to sing the song “Her Lies” exactly like Asaf Avidan, i.e., in a way that is difficult to classify in terms of gender!

To document their engagement, students completed a written reflective exercise via Moodle. They responded to the following two prompts:

(1) Please tell us briefly what you experienced when using the voice apps. You can mention anything that comes to mind, such as it was very strange to hear yourself, I did not feel comfortable with the evaluations or about your experience of trying out a “new” voice. Everything is interesting!
(2) Last but not least, I would like to ask you for a short reflection: To what extent did this course help you to empathize with the lives of trans* people?

Students could voluntarily submit their apps data to enter the *Gendered Voice Competition*, with a CD by Asaf Avidan or Coco Rosie awarded to the strongest performance as rated subjectively by the first author.

### 2.2 Sample

For this study, data were collected on five different dates in October 2025. As the seminar was delivered online, presumably students worked alone with the choice to get synchronous support via Moodle or via Mail.

Overall, 100 undergraduate, fourth-year medical students (of a cohort of 658) participated in the seminar, which was delivered online in five parallel groups of 20 students to ensure a suitable supervisor-student ratio for the “Gendered Voice Competition”. 38 of them identified as male, 62 of them as female, none as non-binary during the application process. Due to the European General Data Protection Regulation (GDPR) framework, in the educational context of “feminize your resonance!” no further socio-demographic data of students’ gender identity were collected. During the seminars, none of the students openly identified as transgender.

### 2.3 Data analysis

The analysis followed a consensual qualitative research approach (25), emphasizing independent coding followed by systematic consensus-building to enhance analytic rigor. A multi-stage analytic process was employed, including familiarization, descriptive coding, and chronological/contextual mapping. The researchers then independently coded the entire dataset using an open, descriptive coding strategy, generating an initial set of ten codes (e.g., discomfort with own voice, surprise at app feedback, realization of vocal effort), which were provisionally organized into three thematic domains.

Coding discrepancies and conceptual overlaps were examined across three structured consensus sessions, during which two codes were subdivided and subsequently integrated under an umbrella theme, resulting in the distinct narrative stages “the search for the self” and “the search for the other,” while the theme Voice awareness was conceptually refined to implicit voice awareness. Through chronological and contextual mapping of the refined codes, a five-phase learning journey was identified, capturing students’ progression from initial app use to a more nuanced understanding of transgender experience. A final consensus-coding session involving a third expert researcher validated the complete framework, resulting in full agreement on a model comprising three overarching themes structured across five narrative phases, with early-stage reflections characterized by codes such as surprise at app feedback and later phases marked by codes such as expressing empathy for trans people.

ATLAS.ti was used to organize and manage the data.

## 3 Results

The total response count for the first question is *n* = 100, for the second question is *n* = 93.

Our findings reveal that voice awareness develops progressively through a series of transformative phases, culminating in a deeper understanding of transgender experiences. This multi-phase learning journey emerged clearly from students’ engagement with the voice-changing applications.

### 3.1 Key themes in the learning process

Through the analysis, three key themes implicit voice awareness, technology engagement, and sharing transgender experience were identified. These themes were chronologically and contextually linked, forming a five-phase trajectory that describes students’ progression from initial app use to an emerging understanding of transgender experiences.

The first theme, *implicit voice awareness*, corresponds to the first phase and captures students´ pre-seminar awareness of their voice. Its cumulative nature is illustrated in light blue in Table 2.

**Table 2:** Number of respondents according to five-phase learning process along key themes.

Phases	Voice awareness		
	Implicit voice awareness	Technology engagement	Sharing transgender experience
Internal echoes and embarrassment (pre-class)	20 respondents		
Apps as external ears and judges		44 respondents	
Experimentation and play		74 respondents	
Identity exploration and defamiliarization		96 respondents	
Social awareness and empathy			66 respondents

It should also be noted that the responses of a single respondent could correspond to multiple phases. Specifically: 1 respondent mentioned all 6 phases in the quotation; 5 respondents mentioned 5 phases in the quotation; 14 respondents mentioned 4 phases in the quotation; 21 respondents mentioned 3 phases in the quotation; 35 respondents mentioned 2 phases in the quotation; 22 respondents mentioned 1 phase in the quotation; 6 respondents did not mention any phases in the quotation.

Since our distribution is normal, it can be stated that the sample size and the coding were appropriately chosen and done. Furthermore, the exploratory coding was derived from the entire set of respondents. The different nuances of blue represent the numbers of participants who did articulate participation in the respective learning phase, the darker the blue the more participants did so.

“I first tried saying a short text and found it very strange to hear my own voice, as I had always thought it was deeper. I also don’t like listening to audio of myself because I find it alienating. Then I tried singing a bit and adjusted my voice with the tools; I found it interesting how different I sounded and how unrecognizable I became to myself” (P6).

“It generally feels strange to hear your own voice. When it’s also modified, it’s even weirder, because I think you can never *not* notice the difference. I wonder if that changes over time (i.e., if you eventually are no longer surprised when you hear your “modified voice” in videos)” (P8).

The second theme, *technology engagement*, captures how students expanded their awareness of voice-its variability, measurability, and social meaning-through three consecutive phases.

“Although I’ve heard my own voice on recordings many times, it’s always a bit strange. It was very interesting to experiment with the app; to try speaking with a higher or slightly deeper voice and to test out more feminine speech melodies” (P37).

“It was interesting to see that, as a woman, I was always in the “female” zone. No matter how hard I tried, I could hardly, or not at all, reach the “male” zone (e.g., by using a deeper voice, a more voluminous voice, etc.)” (P21).

“It was unfamiliar to hear myself with a modified voice. The analyses were confusing at first, but it was exciting to see how my voice can change. Speaking in a higher register was strenuous, but motivating and inspiring; I felt like I was getting closer to a “new” voice. Gender-based changes were also very interesting” (P96).

The final theme, *sharing transgender experience*, reflects the phase in which students connected their newly gained voice awareness to broader questions of gender incongruence and the everyday challenges faced by transgender people.

“I worked with the Voice Tools app and was really impressed at how clearly the differences in vocal pitch were visible. A friend was with me, and it was fascinating to see that we actually speak in completely different pitches—I had honestly never thought about that before. Through this experience, I can now better understand that people with atypical vocal pitch may face difficulties, which can affect their quality of life as well as their sense of social belonging” (P87).

“I hadn’t realized before how many limitations this can cause in everyday life – for example, that some trans* people avoid speaking in public to prevent uncomfortable situations or discrimination. The course encouraged me to be sensitive and attentive when interacting with trans* people and to pay more attention to their individual needs” (P26).

“Since I had never thought about how the voice contributes to the well-being of a trans person, this course opened my eyes to the topic. I had also never heard of voice apps before, and I think they are a good way to support the well-being of trans people” (P38).

As these excerpts illustrate, the developmental process established during the seminar Feminize your resonance! reflects Kolb’s (26) experiential learning cycle, while adding unique dimensions related to gendered voice. Table 2 summarizes the non-linear progression from vocal self-awareness to transgender advocacy across the five phases learning journey.

The first theme, implicit voice awareness, corresponds to the first phase and captures students´ pre-seminar awareness of their voice. Its cumulative nature is illustrated in light blue in Table 2.

### 3.2 Key theme 1: implicit voice awareness

Students exhibited a form of implicit voice awareness before using the app. Phase 1, internal echoes and embarrassment, captures this initial, largely solitary engagement with their own recorded voice. This experience was shaped by internal judgment rather than critical reflection or external input.

#### 3.2.1 Phase 1: internal echoes and embarrassment (pre-class)

Before the seminar, students were familiar with hearing their recorded voices through platforms such as WhatsApp, often adjusting playback speed or re-listening. However, evaluations of their voice in this phase were internal and frequently marked by discomfort or avoidance of auditory self-perception. As one student notes:

“I generally find it very uncomfortable to hear my own voice and am even surprised that it’s supposed to be me when I hear it” (P14).

This aligns with “voice confrontation anxiety” (27), describing the dissonance between internal and external vocal images. These pre-existing experiences created a baseline readiness for more structured reflection during the seminar.

### 3.3 Key theme 2: technology engagement

This theme encompasses students´ encounters with TMF, moving from playful experimentation to critical analysis and personal reflection. For some, the app remained primarily a tool for gaming or entertainment, reflected in the experimentation and play phase. Others, however, moved beyond this initial curiosity and began to explore the app’s functions more deliberately. In doing so, they developed greater awareness of their own vocal patterns as well as a growing understanding of the challenges that voice modification poses for others, particularly for transgender individuals. This deeper engagement becomes especially visible in the identity exploration and defamiliarization phase.

#### 3.3.1 Phase 2: apps as external ears and judges

With the introduction of the app, voice evaluation shifted from internal to external. The application assigned quantitative labels-pitch values, gendered classifications-and students reacted with surprise, curiosity, or amusement. The perceived objectivity of the algorithm enhanced the emotional impact of the feedback:

“I really enjoyed getting feedback on my own voice. As I spent more time with the app, I had fun trying to achieve changes” (P42).

The app’s metrics rendered gendered vocal norms visible, supporting students to recognize the measurable dimensions of voice incongruence.

#### 3.3.2 Phase 3: experimentation and play

Many students initially approached the app as a novelty or game, exploring how changes in speed altered measured outcomes. As a student mentions: “When I tried one of the voice apps myself, it was initially quite unusual, but also very exciting to receive immediate feedback. This made it possible to try out different methods and observe how the voice was affected” (P38).

For some students, this exploratory stage served as a gateway to deeper engagement for some students.

#### 3.3.3 Phase 4: identity exploration and defamiliarization

When prompted to imitate a different gendered voice or to speak in a non-native language, students began to step outside established vocal habits. This elicited moments of defamiliarization and initiated more reflective engagement with their own voice and vocal identity.

Within this phase, students progressed through three sub-movements:

Phase 4.1 system analysis: some students mention, “It is interesting that the classification is based solely on frequency or Hertz, because in my opinion, when you actively modify your voice into a different frequency range, you notice that the voice is being artificially altered. At the same time, I can easily imagine that if you didn’t know the voice was modified, it would be very difficult to correctly identify such a vocal alteration. It is sometimes very hard to distinguish between a male and a female voice. I think that in real life, a natural bias is created when you can assign a voice to a person” (P9). Or say “My experience with the voice app confirms the central insight that voice satisfaction does not depend on technical ideal values. My voice frequency is rather in the lower female range. So, I would argue that successful voice adaptation is achieved when the voice authentically reflects gender identity, regardless of specific frequency ranges” (P50). These Students are actually critiquing the app’s binary, frequency-based model, recognizing its limitations and its constructed nature:

Phase 4.2 Search for the self: students turned the technology inward, confronting discrepancies between internal self-perception and external recordings. This is the step reflected in comments such as “I would only have been able to identify it as 90% myself” and “I found it interesting how different I sounded and how unrecognizable I became to myself”. The realization that one’s voice fluctuates with mood-described, for example, as “more feminine/ higher… motivated, happy”-emerged as a key insight, highlighting voice as a dynamic expression rather than a fixed characteristic. As one student notes: “Using the Voice Tools app, I personally found it very interesting to see how my own speech was divided into categories”. As a cis person, one usually does not think about their own speech or how others perceive it. Through the app, I could see how my voice was categorized as male versus female, and I could specifically experiment with how conscious changes affected it” (P54).

Or another one says: “Experimenting with the app was more enjoyable than expected, and I found it fascinating to learn something about my own gendered voice qualities, volume, and frequency” (P60).

Phase 4.3 Search for the other: with a critical understanding of the technology and their own vocal struggle, some students used this experience as an empathetic bridge to understanding others. Comments such as “it’s quite exhausting to raise one’s pitch without it sounding artificial” and “how difficult it is to change one’s voice” illustrate this search for the other. The recognition that different voices are associated with “completely different types of people” reflects students´ growing awareness of how vocal gender perception operates within society, and allowed to gain insights in the struggles of transgender individuals. Some students describe this shift as follows: “When using the voice apps, I first found it strange to hear myself. Experimenting with new voices showed me that one’s voice contributes much more to who one is than one usually realizes. I became aware of how much it distinguishes a person from others and how unique each voice is. I can also now better imagine how unusual it must feel for transgender people when their own voice suddenly sounds different” (P20).

Some students even proposed clinical uses for such apps: “Working with voice apps was both helpful and unfamiliar. It was particularly strange to hear my voice in recordings, as it sounds very different from how I perceive it while speaking. This discrepancy between self-perception and external perception takes some getting used to, but also offers an intriguing opportunity to observe the voice more objectively. Precisely because of this, I realized how useful such apps can be for documenting one’s progress. Especially for trans* women, these recordings can provide a valuable starting point for consciously tracking changes during voice training” (P25).

### 3.4 Key theme 3: sharing transgender experience

Key Theme 3 involves the emerging understanding of others´ lived realities: students, now, are able to relate gender, voice, and identity and gained respect for transgender individuals.

#### 3.4.1 Phase 5: social awareness and empathy

In the final phase, students articulated a clearer understanding of the challenges transgender individuals may encounter while aligning voice and gender identity. The experiential component facilitated affective learning: “I find it strange to hear my own voice as a recording. I think many people find this weird. And that makes me think: if I find it strange, even though my voice in the recording isn’t completely different from my self-perception, then it must be really difficult for trans* people to feel good about their voice, their self-perception, and their gender identity, or to feel like themselves” (P75).

Phases 4 and 5 can be understood as phases of detachment (from habitual vocal identity) and reattachment (to new social and embodied awareness).

### 3.5 The affective learning process

Table 3 illustrates the affective learning process, presenting the different descriptive adjectives for how students felt during single phases. Students’ emotions in the first phase are characterized predominantly by negative emotions located within Plutchnik’s (28) basic emotion clusters annoyance-anger-rage and boredom-disgust-loathing, which together form the primary dyad emotion ‘contempt’. However, there are also emotions present, located in the positive cluster distraction-surprize-amazement.

**Table 3:** Affective learning process: feelings used in students’ encounters.

The phase	The feeling	Sample comment
Internal echoes and embarrassment (pre-class)	Strange, alienating, weird, uncomfortable, surprised, dislike, extremely unpleasant, odd, terrible	I personally always find it a bit strange to hear my voice on a recording. I know many people feel the same way, who, for example, find it “totally weird” to listen to their own voice messages.
Apps as external ears and judges	Fascinating, interesting, insightful, unfamiliar, helpful, unusual, exciting, confusing, surprised, skeptical	I found it fascinating how one can analyze and deliberately modify one’s own voice. I also found it interesting to see how you can measure things like pitch or volume in real time. It does not replace professional guidance, but as an introduction, I found it quite insightful.
Experimentation and play	Interesting, exciting, confusing, helpful, strange, fun, curious, uncertain, surprised, awkward, unfamiliar, enjoyable, strenuous	I found it very interesting to see the position of my own voice within the spectrum of “gender ranges” and to observe how the pitch changes when pronouncing different sentences.
Identity exploration and defamiliarization	Instructive, interesting, surprised, exhausting, unnatural, fascinating, suspected, extremely difficult, interesting, exciting, aware, conscious, exhausting, motivating, inspiring, intriguing	At times, it was difficult to understand exactly what the results meant, but it was interesting to experiment with making my voice sound “more feminine” or “more neutral”. Overall, it helped me become more aware of how I use my voice.
Social awareness and empathy	Reflective, moved, empathetic, understanding, admirable, respectful, sensitized	It helped me to some extent to put myself in the shoes of transsexual people. It draws attention to a challenge this group faces that one would otherwise never think about—and never have to think about if not personally affected. It’s also a problem that is anything but easy to solve, since surgeries come with many risks and various voice trainings often do not lead to the results trans people hope for. It made me reflect more broadly on the many other issues transsexual people face—issues I had never really thought about before.

In the fifth phase the picture is different: The students’ emotions are characterized by predominantly positive emotions located in the basic emotion clusters interest-anticipation-vigilance, serenity-joy-ecstasy and acceptance-trust-admiration, which combine to the primary dyads ‘optimism’ and ‘love’.

## 4 Discussion

This study hypothesized that employing voice-modulation technology within medical education can foster students’ empathizing with transgender individuals by illuminating the centrality of voice for health, identity, and social participation. The narrative analysis supports this proposition: engaging with voice-changing software provided a pedagogical space in which students could confront their own assumptions about vocal identity and gradually extend this awareness toward the lived realities of transgender persons. The emergent five-stage engagement model-ranging from self-confrontation to social empathy-offers a replicable and transferable framework for teaching the entanglements of voice, identity, and health while strengthening learners´ capacity to empathize with transgender individuals. Concluding, the journey through detachment and reattachment-induced by TMF- transformed students from individuals who have a voice to individuals who understand the voice as central to the construction of self and social recognition.

The intervention’s critical function lay in making students’ previously implicit vocal identity explicit, and thus accessible for reflection. This moment of defamiliarization encouraged learners to reconsider the voice not as a fixed biological property but as an intentionally mutable expression of the lived body, intimately tied to self-understanding and social legibility. Such experiential insight resonates with phenomenological accounts that distinguish between the clinician’s externalized perspective of the body and the patient’s subjective embodied experience (29, 30). Approaching voice through this dual lens underscores why vocal transition is not merely a technical adjustment but a form of embodied labor embedded in gendered social worlds.

The rapid shift from heightened self-consciousness to outward-directed empathy demonstrates the potential of targeted, technology-supported teaching formats to enhance diversity in undergraduate medical curricula. These findings are consistent with research on embodied simulation, which indicates that experiential engagements deepen cognitive and affective learning, especially in areas where students may initially lack familiarity or confidence (31, 32). The strong empathetic responses documented here-including students´ recognition of the challenges associated with vocal transition-reinforce recent calls to integrate “affective competencies” more systematically into medical training (33), particularly in relation to marginalized patient groups.

Future longitudinal research should not only examine whether such early technology-based educational encounters promote empathy towards transgender individuals but also whether familiarising students with state-of-the-art practice in voice therapy translates into sustained shifts in clinical practice. We would also like to point out that one potential success factor in our setting may have been that the technology-supported activity was delivered by a certified speech instructor. This is important because, in clinical practice, technological tools are viewed as complementary support to the behavioral interventions delivered by speech-language pathologists (SLP). With an SLP or a speech instructor acting in the role of facilitator, it is ensured that the complementary nature of technology in voice training is conveyed to students.

Tracking former students into postgraduate clinical training and early professional practice could help determine whether exposure to voice-related embodied learning is associated with improved patient-centered communication, reduced bias, or better health for transgender individuals. Such evidence would strengthen the case for integrating voice-focused experiential modules into broader strategies aimed at reducing health disparities.

## 5 Limitations

However, our conclusions must be tempered by the study’s limitations. Self-reported data, collected within a graded, compulsory-elective course, may have constrained the openness with which students articulated their experiences. Also, given the study setting within the official curriculum, it was not possible to inquire about students’ gender identity beyond what is available in official student records. There may also be a societal issue related to the disclosure of non-binary gender identity. Compared to the estimated 0.3–0.5% of the adult transgender population (7), the proportion of students identifying as non-binary in Austria appears higher: in a national anonymous student social survey including 43,376 individuals seems 1% declared themselves as non-binary and 0.6% chose not to declare their gender (34). However, although non-binary identification options are also offered during the medical school application process, they are rarely used: Amongst 5,332 applicants for human medicine, only five selected a non-binary gender category (MedAT 2024, unpublished internal report). A similar study conducted in an experimental setting outside the formal curriculum, where anonymous participation might be possible, may therefore be a next step in more closely describing the effects of these interventions.

The single-institution nature of the study, its western context, and the inclusion of undergraduate medical students only limit the generalizability of the findings. The core problem – undergraduate students being unfamiliar with transgender individuals and consequently holding stigmatizing attitudes that may lead to discrimination in health care – has also been identified as relevant in non-western contexts, for example among speech and language pathology students in South Asia (35).

While in principle, the educational strategy of using experiential engagement with voice technology to support transformative learning may be transferable to other cultural contexts, careful consideration is required which voice applications with TMF are used and how experiential learning is facilitated to ensure appropriateness for the respective cultural setting.

The app’s technical parameters-particularly the absence of resonance-related metrics-reflect wider shortcomings in voice-modulation tools designed for gender-diverse populations. These technical and contextual constraints may have shaped the depth of students´ learning as well as the kinds of narratives they produced. However, given the richness of accounts provided by our sample, we consider this limitation as minor.

Future iterations of the intervention would benefit from structured intersubjective feedback from transgender community members and clinicians specializing in transgender voice work. Such collaboration could help ensure that pedagogical uses of voice technology align with clinical needs, avoid reproducing algorithmic biases, and more accurately convey the nuance involved in vocal transition.

## 6 Conclusion

“Feminize Your Resonance!” demonstrates how experiential engagement with voice technology can bridge the gap between theoretical knowledge and compassionate, gender-affirming healthcare. By rendering the often-invisible labor of vocal gender expression tangible, this intervention equips future clinicians to appreciate the complexity of voice in transgender wellbeing and to advocate for non-invasive, accessible interventions. Encouraging learners to “walk in the shoes” of their future patients through embodied simulations may also contribute to reducing stigma and supporting the de-pathologization of non-binary and transgender identities, who are reported to experience discrimination in public and professional life.

## Data Availability

In accordance with institutional data protection regulations, the raw data for this study cannot be shared with parties outside the institution.
